# Predictive Technologies: Can Smart Tools Augment the Brain's Predictive Abilities?

**DOI:** 10.3389/fnins.2016.00186

**Published:** 2016-04-27

**Authors:** Giovanni Pezzulo, Alessandro D'Ausilio, Andrea Gaggioli

**Affiliations:** ^1^Institute of Cognitive Sciences and Technologies, National Research CouncilRome, Italy; ^2^IIT - Istituto Italiano di Tecnologia, CTNSC@UniFe - Center of Translational Neurophysiology for Speech and CommunicationFerrara, Italy; ^3^Department of Psychology, Università Cattolica del Sacro CuoreMilan, Italy; ^4^Applied Technology for Neuro-Psychology Lab, I.R.C.C.S. Istituto Auxologico ItalianoMilan, Italy

**Keywords:** predictive processing, planning, robotics, augmented reality, brain stimulation

## Abstract

The ability of “looking into the future”—namely, the capacity of anticipating future states of the environment or of the body—represents a fundamental function of human (and animal) brains. A goalkeeper who tries to guess the ball's direction; a chess player who attempts to anticipate the opponent's next move; or a man-in-love who tries to calculate what are the chances of her saying yes—in all these cases, people are simulating possible future states of the world, in order to maximize the success of their decisions or actions. Research in neuroscience is showing that our ability to predict the behavior of physical or social phenomena is largely dependent on the brain's ability to integrate current and past information to generate (probabilistic) simulations of the future. But could predictive processing be augmented using advanced technologies? In this contribution, we discuss how computational technologies may be used to support, facilitate or enhance the prediction of future events, by considering exemplificative scenarios across different domains, from simpler sensorimotor decisions to more complex cognitive tasks. We also examine the key scientific and technical challenges that must be faced to turn this vision into reality.

## Introduction

Modern cognitive neuroscience describes the brain as a predictive device, not a stimulus-response system. In this “predictive brain” perspective, the brain continuously predicts environmental dynamics and anticipates action effects, and this permits animals to be “ahead of time” when it takes decisions, rather than just react to what it currently senses (Pezzulo, 2008; Bar, 2009; Friston, 2010). Predictive abilities range from short-term predictions, which target (say) the few hundreds of milliseconds—the timescale of the sensorimotor loop, which is relevant to predict (say) whether or not to cross a busy road—to medium- or long-term predictions, which are useful for distal decisions such as the road to take to go home or the career to choose.

Recent research is shedding light on the neuronal underpinnings of these diverse abilities. For example, a growing literature studies predictive dynamics in sensorimotor control, highlighting the importance of so-called forward models: internal models that the brain uses to predict action consequences and plan accurate movements (Wolpert and Ghahramani, 2000). Even more interesting, research in “motor cognition” has shown that these forward models can be widely reused outside motor control, in more cognitive such as domains action recognition, action simulation and imagery (Jeannerod, 2006). Predictive domains, have been widely studied in many other domains of cognition, such as perceptual processing and navigation. For example, growing evidence indicates that internally generated brain dynamics in the (rodent) hippocampus might support navigational planning: in fact, if one registers from the hippocampus of rodents at rest before a decision, one can find neuronal sequences of place cells assembled to form trajectories in T-mazes, which can be predictive of the actual trajectories that they will select immediately afterwards (Johnson and Redish, 2007; Pfeiffer and Foster, 2013; Pezzulo et al., 2014). Based on parallels between neurophysiological evidence and human neuroimaging studies, it has been (speculatively) proposed that the ability to internally generate and “mesh” dynamical events—especially in the hippocampus—might support sophisticated prospective abilities such as the mental simulation of future events and “mental time travel” (Schacter et al., 2012; Buzsáki et al., 2014).

From the “predictive brain” perspective, the ubiquity of predictive components across cognitive domains reflects the fact that brain processing is intrinsically predictive. The most comprehensive attempt to describe formally the “predictive brain” perspective is the *free energy principle* developed by Friston and collaborators (Friston, 2010; Pezzulo et al., 2015). In this perspective, the brain is a statistical machine that learns a so-called *generative model* of external dynamics (especially how the environment changes as a function of the agent's actions) and uses it for continuous prediction. Importantly, prediction can be at multiple timescales, where these timescales map to brain hierarchies (especially cortical hierarchies) and increase from motor and premotor areas to prefrontal areas (Badre, 2008). Here, cognition depends on the interplay of top-down and bottom-up signals across (brain) hierarchical layers, the former propagating predictions and the latter prediction errors. Minimizing prediction errors (the difference from what is expected and what is sensed) across layers—or more formally minimizing free energy—supports both perception (because perceptual hypotheses encoded at higher levels can be revised based on prediction errors) and action (because goals encoded at higher hierarchical layers generate a cascade of exteroceptive and proprioceptive predictions, say on the next desired hand position, and the latter are ultimately suppressed by engaged reflexes that—essentially—guide the hand to the desired position). The same principles of prediction error minimization have been extended to model the planning of action sequences—when, essentially, predictions stemming from the generative model (encoding the agent's knowledge of action-outcome contingencies) are “chained” to covertly simulate and evaluate possible action plans in advance, before the agent performs any actual action or receives external feedback (Friston et al., 2012, 2015).

In sum, the idea of a “predictive brain” is becoming dominant in cognitive neuroscience. In this article, we explore an intriguing technological side of this phenomenon: the possible development of “predictive technologies” that augment human prediction. We address questions such as: is it possible to augment predictive abilities in humans using technologies? Which kind of predictions can be enhanced? Is it possible to use enhanced predictive abilities to improve real-time decisions and actions? What are the key scientific/technological issues for developing predictive technologies? And what are their potential applications?

Of course, prediction-based technologies are already routinely used, for example, in weather or stock market forecast, or in the development of smart (e.g., self-guiding) cars. Here, however, we specifically focus on *predictive technologies that can be seamlessly integrated into real-time human cognition, and augment it*. We discuss, for example, new interfaces that help humans predict the movements of a car in order to decide when to cross a busy road. Below we consider this and other specific examples of predictive technologies that—we will argue—might be soon within our reach.

## Domains of enhanced prediction

Human cognitive tasks involve short-, medium- or long-term predictions—and at least in principle, predictive technologies can support all them. Here we focus our analysis on predictive technologies that can support real-time, embodied decisions (Cisek and Pastor-Bernier, 2014; Lepora and Pezzulo, 2015), such as for example those that we continuously face when we drive a car (should I accelerate or press the brake?), which are based on short-term predictions of the order of hundreds of milliseconds (am I risking a collision with a pedestrian?) or sometimes medium-term predictions; but we do not target decisions that unfold over longer-term timescales such as “which University or career should I select?”.

It is important to distinguish between two potential outputs of predictive technologies. The first, direct output of a predictive technology is enhancing a person's prediction abilities. For example, the technology might help a goalkeeper predicting the trajectory of a penalty kick, on the basis of (say) statistical information about past penalty kicks and the movements of the attacker. A second, more indirect output of predictive technologies is aiding decision-making and/or planning (based on prediction). For example, the predictive technology might suggest the goalkeeper the action course that maximizes the probability to parry the penalty kick, given the predicted trajectory. Below we provide examples of both kinds.

Consider the case of a person who wants to cross a busy road. Deciding when to cross is a complex embodied decision that has to take into consideration several factors, from cognitive rules and knowledge (e.g., knowledge of traffic rules; presence of traffic lights) to situational facts (e.g., where are the cars and at what velocity they run; what my own velocity is; how long the road to be crossed is). Various predictions can be useful to solve this task, such as the prediction of “where” a car is going, “whether” it will stop or “when” it will arrive at the crossing point. There are cases when an augmentation technology may help, such as when the person who is crossing has impaired perceptual or cognitive abilities, or comes from another culture (think of a Chinese tourist crossing a busy road in Rome).

**Figure 1:**
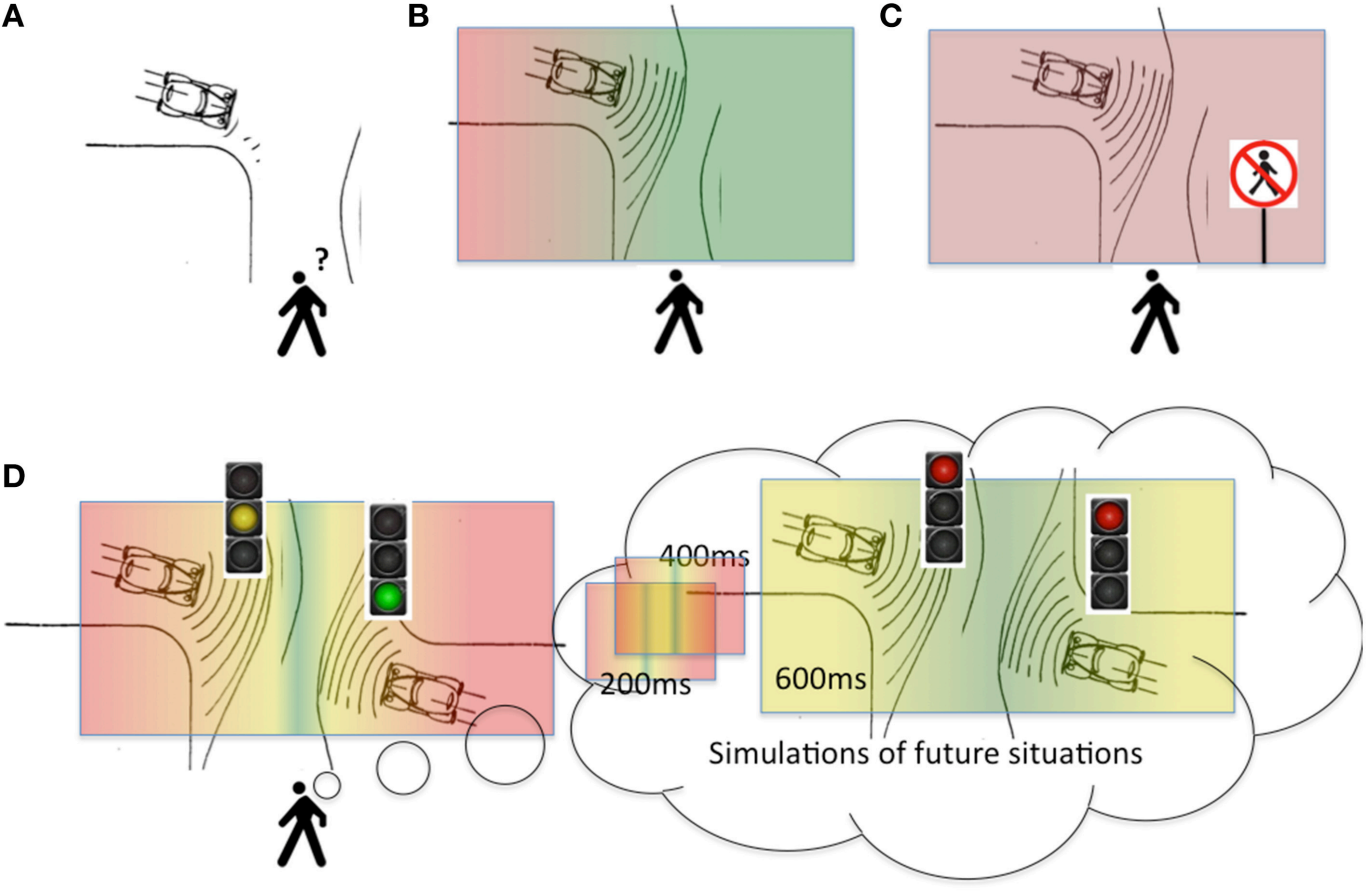
**How the affordance landscape changes as an effect of car movements and traffic signs. (A)** An example situation: a pedestrian has to cross a road, with a car approaching. **(B)** The same situation, but now with the putative results of a predictive technology superimposed: here, the lines in front of the car represent its predicted future locations, and the colors represent a gradient of the “cross-ability” affordance (red = not crossable; green = crossable) ordered in a continuum. **(C)** The same situation with a traffic panel does not afford cross-ability anymore. **(D)** A more complex scenario, in which the predictive technology might simulate future simulated situations, e.g., display how the affordance landscape will change in the next 200, 400, and 600 ms.

How can we design a predictive technology that augments human cognition in this kind of situation? A useful starting point is the notion of an “affordance,” which originates from ecological psychology (Gibson, 1979) but is also used in neuroscience (Cisek and Kalaska, 2010; Pezzulo and Cisek, 2016). In this perspective, an empty street *affords* “cross-ability,” whereas a busy road does not. More generally, one can think of a “landscape” of affordances, which changes as an effect of environmental dynamics and our own actions. For example a car approaching fast in our direction (see Figure 1) modifies the landscape of “cross-ability” over time: when the car is far, the road is cross-able; but when it approaches, it becomes not cross-able. The concept of a landscape of affordances is dynamical and highly context-sensitive, in the sense that (for example) there is a gradient of “cross-ability” over time and it depends on both our and the car's velocity and direction, as well as the color of the traffic light (see Figures 1B–D).

**Figure 2:**
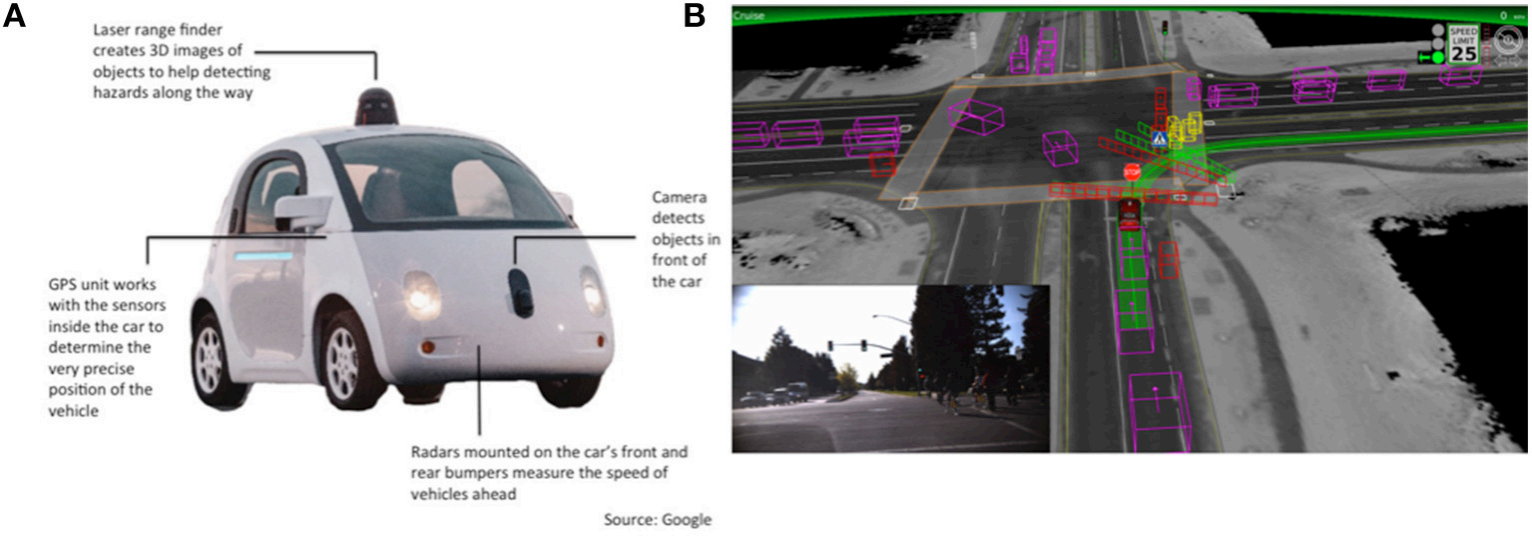
**Examples of enabling technologies**. **(A)** Sensors of a self-driving car (source: Google). **(B)** Schematic of how objects are represented (from the self-guiding car's perspective) while approaching a turn; the inset shows what a human driver sees from inside the car. This representation can be used to generate predictions (e.g., of the trajectory of cars and pedestrians) based, for example, on probabilistic mechanisms (Thrun et al., 2006).

While ecological psychology has often focused on the idea of exploiting available affordances (e.g., use the “sit-ability” of a chair to sit down), here we recast this concept in predictive terms, and discuss how the *prediction* of (for example) car movements permits to foresee how the affordance landscape will change in the immediate future - and to spot in advance the right time window to cross the road. Figure 2B exemplifies the potential benefits of a predictive technology: an augmented reality display that shows (predictively) the affordance landscape and signals that a good time frame for crossing the road is approaching (see later for a discussion of the feasibility of this technology and its required components). Coming back to our previous distinction between “prediction” and “prediction-based decision or planning,” here the curved lines around the car represent its predicted future locations, while the colored parts represent the future predicted affordances (red = not crossable; green = crossable)—which can be considered as an aid to the decision (when and where to cross) based on the predicted car trajectory.

Another, more complex example concerns the decision of which path to take to cross a crossroad and reach a supermarket. This can be considered analogous to the “travel planning” functionality of Google maps (https://www.google.it/maps), which already takes several factors into account (e.g., length, size of streets, current traffic) to provide an estimate of “the best” or “the faster” road. Predictive technologies can extend these functionalities by considering various contextual factors, such as the current “cross-ability” affordances (as above) and the anticipated effort associated to the different paths (e.g., one path might require climbing stairs)—which in turn might require predicting energy expenditure based on physiological signals (e.g., heart rate) or other information (e.g., age). Here, different from the former example, the predictive technology would not (only) show a field of affordances, but—like Google maps—propose several paths and suggest the best one. Importantly, this exemplifies a medium-term decision, not a short-term decision as in road crossing case, and it can incorporate various elements and various predictions, at short and medium terms.

As a third example, let's consider a professional domain: an aircraft pilot who has to decide which buttons to press, which levers to pull, which indicators to look at, etc. A predictive technology might help pilots predict (for example) the outcome of different maneuver by directing attention to the controls that are likely to show the most critical information. These functionalities might be useful for pilots but more critically to those who are learning to fly; the latter might learn faster if their attention is directed to the most relevant displays or controls.

As these examples illustrate, predictive technologies can augment human predictive abilities and be flexibly incorporated into human cognition, providing raw predictions (of car movements), suggestions (of a road to take) or guiding attention (to the most-likely-to-be-useful displays)—just to make a few examples.

## Enabling technologies

In the previous section we have briefly discussed a few examples of predictive technologies and their use in everyday life. These examples are (on purpose) beyond the scope of the current state of the art. However, some components required by predictive technologies—software or hardware—are already available as components of existing technologies, or are currently studied as part of research programs. Here we provide some examples of enabling technologies that might support the future development of predictive technologies.

Self-driving cars are under development by many companies (e.g., Google, Tesla) and they might enter in the market in the near future. Self-driving cars already use many technologies—from sensors such as laser cameras and radars to dedicated processors—that can be integrated in future predictive technologies, see Figure 2A. Furthermore, at the software level, self-driving cars use prediction for control and planning—for example, to predict car trajectories that avoid collisions; and some of the solutions implemented in self-driving cars might be reused within human augmentation systems such as those that we described in the previous section.

Although self-driving cars use a range of diverse mechanisms, some solutions to key problems such as self-localization, path planning, data fusion and trajectory prediction are reused from robot navigation (Thrun et al., 2006) and most can be solved using probabilistic prediction methods (Thrun et al., 2006). Predictive mechanisms, and in particular methods for approximate-but-fast prediction figure prominently in both robot navigation (Montemerlo et al., 2002) and self-driving cars.

Another example of predictive algorithm that was initially developed for robotics but might be reused (or modified) within predictive technologies is the “internal world model” used by Ripley the robot (Roy et al., 2004). The system uses a physical engine to simulate or predict the robot movements, and to keep the “world model” of the robot updated—for example, to keep simulating the trajectory of a moving ball even if the robot is looking somewhere else. In this way, the robot's internal model is continuously “in register” with the external world. This software exemplifies the idea of using a physical engine—of the kind used, for example, in computer games—to run physically realistic simulations. Many widely-used physical engines are very accurate and can afford real-time simulation or prediction. Of course, they cannot simulate everything with the required level of accuracy (and in real time), thus their putative role within a predictive technology can only be evaluated case-by-case.

Physical engines have been also used for simulation in a series of studies on human physical scene understanding, such as how well humans predict how towers of wooden bricks will fall down (Battaglia et al., 2013). This study illustrates that a physical engine simulation can be used to predict in real time a challenging physical event that derives from the interaction of many components (here, many wooden bricks). This method might be extended to predict how other physical events (e.g., movements of objects such as cars) unfold in time.

Another example of a computational solution developed within robotics is the idea of “affordance gradients”: a formalism that describes the transformations that can be applied on an object (e.g., how a triangular object rotates when it is pushed or pulled) and permits a robot to plan actions that exploit the affordances to achieve goals (e.g., plan a series of pushing actions that guide the triangular object to a desired or goal position, see Sanchez-Fibla et al., 2011). This method can be potentially reused outside robotics to represent e.g., the affordances of objects and infer their dynamics.

As these examples demonstrate, some component solutions of predictive technologies are available or under development. At the same time, these are still incomplete solutions to the problems of prediction, planning and decision, especially in real time (Geffner, 2013; Donnarumma et al., 2016). Thus, a key challenge for future research is integrating and extending these (and other) models to deliver predictive technologies that are effective and usable. Another key issue is making predictive technologies *usable*—a theme that we explore next.

## Representing predictive information

A key challenge of predictive technologies is how to represent the information about an upcoming event (predictive cue). As suggested earlier, augmented reality may provide a viable solution. An augmented reality system allows superimposing digital information on the physical environment in real-time using a smartphone or head-mounted see-through displays coupled with a wearable computer. Thanks to recent progresses, current augmented reality systems afford real-time applications in which the user can interact with synthetic objects, manipulate them and receive additional information about the environment or the task at hand, in the form of images, text, video, audio components, etc.

Predictive information could be represented in augmented reality using various levels of abstraction, ranging from analog models to symbolic cues. For example, consider the question of how to represent the predicted trajectory of a ball. One could either visualize a virtual ball that precedes the actual ball (analog representation) or use a dynamic arrow (symbolic representation). In choosing the most appropriate representation of the predictive cue, a key requirement is the definition of temporal constraints. If the augmentation cue is too complex, its processing time may even exceed the timeframe available to complete the task, thus rendering the augmentation useless. For example, in rallying the pacenotes are a commonly used method of describing a route to be taken, in terms of turnings, junctions, the degree and severity of bends etc. The notes are designed to help the driver anticipating the conditions of the course ahead, but with a fast-moving vehicle, they are encoded to carry maximal information in minimum reading time. Relatedly, the design of AR interface should take into account user's cognitive requirements, i.e., by preventing split-attention between multiple predictive cues while preserving global situation awareness. This translates to the need for optimizing the trade-off between cue's maximal informativity about upcoming event(s) and least cognitive processing effort.

A further issue is whether, and in which ways, the presentation of the predictive cue could affect the course of the predicted event. For example, if a goalkeeper is shown the prediction that the penalty taker is going to place the ball at the lower right corner, he/she will jump as fast as possible to the lower right corner in order to reach the ball. However, if the goalkeeper initiates the movement too early, the penalty taker may notice the goalkeeper's intention and kick the ball in the opposite corner. Thus, in designing augmented predictions, it is necessary to model the complex interdependencies between the user's action and the context of his/her action, in order to prevent the predictive cue triggering behaviors that affect the course of events in (unpredictable) ways.

## Conclusions

We have explored the idea of using technology to augment the human ability of predicting future events, by seamlessly integrating anticipatory information in the perception-action loop. The presentation of predictive cues is meant to facilitate perceptual, decision and action processes. Some key building blocks of predictive technology are available from developments in robotics and machine learning. In these fields, the need of accurately modeling the evolution of complex system has led to the deployment of computational solutions that could be eventually re-adapted to match the requirements of predictive cognition. However, the provision of external cues may not be the only possible approach to augment predictive processing. A rather more ambitious strategy could be to directly stimulating brain areas that are implicated in the computation of future events. Numerous studies have shown the possibility of modulating, and in some cases enhancing, cognitive processes by exciting brain regions involved in working memory and attention through transcranial electrical brain stimulation, including planning ability (Dockery et al., 2009). This approach could be justifiable within rehabilitation domains, where the goal would be to restore or support predictive functions in individuals suffering from neurocognitive impairments.

As the rehabilitation example suggests, predictive technologies hold the promise to enable a wide range of applications based on the extension of our prediction and planning abilities. Such application scenarios may include, but are not be limited to, anticipation of sources of dangers in natural or working environments for enhancing personal safety; support decision making and judgments in emergency situations; optimization of team coordination in complex collaboration tasks. Clearly, the exploitation of these possibilities require to address significant scientific and technological challenges, some of which have been outlined in this contribution. However, as understanding of cognitive mechanisms involved in predicting future progresses, so should the ability of enhancing these processes using advanced technologies.

## Author contributions

GP and AG jointly conceived the main idea behind the article. GP drafted the manuscript. AD and AG gave conceptual advice and revised the manuscript critically for important intellectual content. GP and AG edited the manuscript. All authors read and approved the final manuscript.

### Conflict of interest statement

The authors declare that the research was conducted in the absence of any commercial or financial relationships that could be construed as a potential conflict of interest.

## References

[B1] Badre D. (2008). Cognitive control, hierarchy, and the rostro–caudal organization of the frontal lobes. Trends Cogn. Sci. 12, 193–200. 10.1016/j.tics.2008.02.004 18403252

[B2] Bar M. (2009). The proactive brain: memory for predictions. Philos. Trans. R. Soc. Lond. B Biol. Sci. 364, 1235–1243. 10.1098/rstb.2008.0310 19528004 PMC2666710

[B3] Battaglia P. W. Hamrick J. B. Tenenbaum J. B. (2013). Simulation as an engine of physical scene understanding. Proc. Natl. Acad. Sci. U.S.A. 110, 18327–18332. 10.1073/pnas.1306572110 24145417 PMC3831455

[B4] Buzsáki G. Peyrache A. Kubie J. (2014). Emergence of cognition from action. Cold Spring Harb. Symp. Quant. Biol. 79, 41–50. 10.1101/sqb.2014.79.024679 25752314 PMC4895837

[B5] Cisek P. Kalaska J. F. (2010). Neural mechanisms for interacting with a world full of action choices. Annu. Rev. Neurosci. 33, 269–298. 10.1146/annurev.neuro.051508.135409 20345247

[B6] Cisek P. Pastor-Bernier A. (2014). On the challenges and mechanisms of embodied decisions. Philos. Trans. R Soc. Lond. B Biol. Sci. 369, 20130479. 10.1098/rstb.2013.0479 25267821 PMC4186232

[B7] Dockery C. A. Hueckel-Weng R. Birbaumer N. Plewnia C. (2009). Enhancement of planning ability by transcranial direct current stimulation. J. Neurosci. 29, 7271–7277. 10.1523/JNEUROSCI.0065-09.2009 19494149 PMC6666475

[B8] Donnarumma F. Maisto D. Pezzulo G. (2016). Problem solving as probabilistic inference with subgoaling: explaining human successes and pitfalls in the Tower of Hanoi. PLoS Comput. Biol. 12:e1004864. 10.1371/journal.pcbi.1004864 27074140 PMC4830581

[B9] Friston K. (2010). The free-energy principle: a unified brain theory? Nat. Rev. Neurosci. 11, 127–138. 10.1038/nrn2787 20068583

[B10] Friston K Rigoli F. Ognibene D. Mathys C. Fitzgerald T. Pezzulo G. (2015). Active inference and epistemic value. Cogn. Neurosci. 6, 187–214. 10.1080/17588928.2015.1020053 25689102

[B11] Friston K. Samothrakis S. Montague R. (2012). Active inference and agency: optimal control without cost functions. Biol. Cybern. 106, 523–541. 10.1007/s00422-012-0512-8 22864468

[B12] Geffner H. (2013). Computational models of planning. Wiley Interdiscip. Rev. Cogn. Sci. 4, 341–356. 10.1002/wcs.1233 26304223

[B13] Gibson J. J. (1979). The Ecological Approach to Visual Perception. Mahwah, NJ: Lawrence Erlbaum Associates, Inc.

[B14] Jeannerod M. (2006). Motor Cognition. Oxford: Oxford University Press.

[B15] Johnson A. Redish A. D. (2007). Neural ensembles in CA3 transiently encode paths forward of the animal at a decision point. J. Neurosci. 27, 12176–12189. 10.1523/JNEUROSCI.3761-07.2007 17989284 PMC6673267

[B16] Lepora N. Pezzulo G. (2015). Embodied Choice: how action influences perceptual decision making. PLOS Comput. Biol. 11:e1004110. 10.1371/journal.pcbi.1004110 25849349 PMC4388485

[B17] Montemerlo M. Thrun S. Koller D. Wegbreit B. (2002). FastSLAM: a factored solution to the simultaneous localization and mapping problem in Proceedings of the Eighteenth National Conference on Artificial Intelligence (Edmonton, AB), 593–598.

[B18] Pezzulo G. (2008). Coordinating with the future: the anticipatory nature of representation. Minds Mach. 18, 179–225. 10.1007/s11023-008-9095-5

[B19] Pezzulo G. Rigoli F. Friston K. (2015). Active Inference, homeostatic regulation and adaptive behavioural control. Prog. Neurobiol. 134, 17–35. 10.1016/j.pneurobio.2015.09.001 26365173 PMC4779150

[B20] Pezzulo G. van der Meer M. A. Lansink C. S. Pennartz C. M. (2014). Internally generated sequences in learning and executing goal-directed behavior. Trends Cogn. Sci. 18, 647–657. 10.1016/j.tics.2014.06.011 25156191

[B21] Pezzulo G. Cisek, P. (2016). Navigating the affordance landscape: feedback control as a process model of behavior and cognition. Trends Cogn. Sci. 10.1016/j.tics.2016.03.013 27118642

[B22] Pfeiffer B. E. Foster D. J. (2013). Hippocampal place-cell sequences depict future paths to remembered goals. Nature 497, 74–79. 10.1038/nature12112 23594744 PMC3990408

[B23] Roy D. Hsiao K. Y. Mavridis N. (2004). Mental imagery for a conversational robot. IEEE Trans. Syst. Man. Cybern. B. Cybern. 34, 1374–1383. 10.1109/TSMCB.2004.823327 15484910

[B24] Sanchez-Fibla M. Duff A. Verschure P. F. (2011). The acquisition of intentionally indexed and object centered affordance gradients: a biomimetic controller and mobile robotics benchmark in Intelligent Robots and Systems (IROS), 2011 IEEE/RSJ International Conference on. IEEE, (San Francisco, CA), 1115–1121.

[B25] Schacter D. L. Addis D. R. Hassabis D. Martin V. C. Spreng R. N. Szpunar K. K. (2012). The future of memory: remembering, imagining, and the brain. Neuron 76, 677–694. 10.1016/j.neuron.2012.11.001 23177955 PMC3815616

[B26] Thrun S. Montemerlo M. Dahlkamp H. Stavens D. Aron A. Diebel J. (2006). Stanley: The robot that won the DARPA Grand Challenge. J. Field Robot. 23, 661–692. 10.1002/rob.20147

[B27] Wolpert D. M. Ghahramani Z. (2000). Computational principles of movement neuroscience. Nat. Neurosci. 3, 1212–1217. 10.1038/81497 11127840

